# Supplementary material for: Association of Low-Grade Glioma Diagnosis and Management Approach with Mental Health Disorders: A MarketScan Analysis 2005–2014
Source: Cancers (Basel). 2022 Mar 8;14(6):1376. doi: 10.3390/cancers14061376 (PMC8946211; doi:10.3390/cancers14061376)
Supplement: Supplementary file 1 [file cancers-14-01376-s001.zip › cancers-1571816-supplementary.pdf]

cancers

MDPI

Supplementary Material: Association of Low-Grade Glioma Diagnosis and Management Approach with Mental Health Disorders: A MarketScan Analysis 2005–2014

Debarati Bhanja, Djibril Ba, Kyle Tuohy, Hannah Wilding, Mara Trifoi, Varun Padmanaban, Guodong Liu, Michael Sughrue, Brad Zacharia, Douglas Leslie and Alireza Mansouri*

Table S1. Psychotropic Drugs to Identify Patients with MHDs [43].

Drug Class	Medications
Tricyclic Antidepressants	Amitriptyline<br>Amoxapine<br>Clomipramine<br>Doxepin<br>Imipramine<br>Maprotiline<br>Nortriptyline<br>Protriptyline<br>Tramipramine
Monoamine oxidase inhibitors	Isocarboxazide<br>Phenelzine<br>Selegiline<br>Tranylcypromine
Selective serotonin reuptake inhibitors	Citalopram<br>Escitalopram<br>Fluoxetine<br>Fluvoxamine<br>Paroxetine<br>Sertraline
Selective serotonin-norepinephrine reuptake inhibitors	Desvenlafaxine<br>Duloxetine<br>Milnacipran<br>Venlafaxine
Other antidepressants	Bupropion<br>Mirtazapine<br>Trazodone
Benzodiazepines	Alprazolam<br>Bromazepam<br>Chlordiazepoxide<br>Clonazepam<br>Clorazepate<br>Clotiazepam<br>Cloxazolam<br>Delorazepam<br>Diazepam<br>Etizolam<br>Halazepam<br>Ketazolam<br>Lorazepam<br>Medazepam<br>Nordazepam<br>Oxazepam

	Phenazepam<br>Pinazepam<br>Prazepam<br>Premazepam
Other anxiolytics	Bretazenil<br>Buspirone

Table S2. Anti-Epileptic Drugs Used to Identify Patients with Glioma-Related Seizures.

Therapeutic Spectrum	Medications
Broad Spectrum Seizures	Brivaracetam<br>Felbamate<br>Lamotrigine<br>Levetiracetam<br>Perampanel<br>Rufinamide<br>Topiramate<br>Valproate<br>Zonisamide
Narrow Spectrum	Carbamazepine<br>Cenobamate<br>Eslicarbazepine<br>Gabapentin<br>Lacosamide<br>Oxcarbazepine<br>Phenobarbital<br>Phenytoin<br>Pregabalin<br>Primidone<br>Stiripentol<br>Tiagabine<br>Vigabatrin

43. Maurer, R.; McNutt, S.; Daggubati, L.; Ba, D.M.; Liu, G.; Leslie, D.; Goyal, N.; Zacharia, B.E. Mental health disorders in newly diagnosed non-functional pitutary adenoma under initial observation: An observational cohort study using the nationwide MarketScan database. *Pituitary* **2021**, *25*, 86–91.
